# Gender, racial, and socioeconomic disparities in the management and survival of patients with locally advanced esophageal cancer: a SEER-based study

**DOI:** 10.1007/s00464-025-11951-7

**Published:** 2025-06-30

**Authors:** Ashley Tran, Sharon Shiraga

**Affiliations:** Division of Upper GI and General Surgery, Department of Surgery, Keck School of Medicine of University of Southern California, Los Angeles, CA USA

**Keywords:** Esophageal cancer, Esophagectomy, Disparities, Minimally invasive surgery, Foregut surgery

## Abstract

**Background:**

Esophageal cancer is the sixth most common gastrointestinal cancer in the United States. Treatment for esophageal cancer depends on the extent of the disease but often includes surgery with or without chemoradiation. The aim of this study is to identify possible gender, racial, and socioeconomic disparities in treatment decisions and survival outcomes for patients with locally advanced esophageal cancer.

**Methods:**

Locally advanced (stage IIA–IVA) esophageal cancer cases in adults (age ≥ 20 years) diagnosed between 2012 and 2021 were identified from the Surveillance, Epidemiology, and End Results database. Data regarding patient demographics, treatment strategies, and survival outcomes was collected. Multivariate logistic and Cox regression analyses were performed to evaluate the association between various patient characteristics and management patterns and survival.

**Results:**

A total of 10,823 cases were included in this study. Female (OR: 0.71, p < 0.001), Black (OR 0.33, p < 0.001), and Hispanic (OR: 0.75, p < 0.001) patients were less likely to be recommended surgery for their esophageal cancer. Among patients who were recommended surgery, Black race (OR: 0.062, p = 0.006) and Low SES (OR: 0.662, p = 0.007) were associated with a lower likelihood of surgery being performed. Female (OR: 0.74, p = 0.007) and Hispanic (OR 0.64, p = 0.003) patients were less likely to receive neoadjuvant therapy. Female (OR: 0.82, p = 0.040), Black (OR: 0.63, p = 0.018), and Low SES (OR: 0.734, p = 0.008) patients were less likely to receive adjuvant therapy. Female patients had improved OS (HR: 0.88, p < 0.001) and CSS (HR: 0.85, p = 0.003) whereas Black race (OS—HR: 1.32, p < 0.001, CSS—HR: 1.31, p < 0.001) and Low SES (OS—HR: 1.15, p < 0.001, CSS—HR: 1.17, p < 0.001) were associated with worse OS and CSS.

**Conclusions:**

Significant differences in surgical management, administration of neoadjuvant and adjuvant therapy, OS, and CSS exist based on gender, race/ethnicity, and SES. Further research is needed to elucidate and ameliorate the possible causes of these disparities.

Esophageal cancer is the sixth most common gastrointestinal cancer in the United States, with its incidence steadily increasing over the past few decades [1]. The management of locally advanced esophageal cancer typically involves neoadjuvant chemoradiotherapy followed by surgical resection, which is considered the standard of care [2, 3].

Despite advancements in treatment of esophageal cancer, significant disparities in outcome and survival have been demonstrated based on sex, race and ethnicity, and socioeconomic status [4–6]. The exact causes of these disparities are not well understood but may be due in part to differences in presentation, access to high-quality care, and management strategies. In general, there is consensus emphasizing the need to better understand the structural barriers which contribute to these disparities to ensure equitable care [7].

Therefore, the aim of this study is to analyze gender, racial, and socioeconomic disparities in the management of locally advanced esophageal cancer. By identifying patterns in treatment recommendations, receipt of therapy, and survival outcomes, this study seeks to provide insights that may inform efforts to reduce disparities and improve cancer care for all patients.

## Methods

Information on adult patients (age ≥ 20) locally advanced esophageal cancer was extracted from the Surveillance, Epidemiology, and End Results (SEER) database between 2012 and 2021. For the purposes of this study, cases of locally advanced esophageal cancer were identified by clinical stage at first visit (IIa–IVa) and metastases status (no distant metastases). Only cases of esophageal adenocarcinoma and squamous cell carcinoma were included in this study. Cases missing relevant patient information were excluded.

Data regarding patients’ age, race, ethnicity, median household income, treatment modalities, and survival were collected. Treatment modalities included neoadjuvant and adjuvant chemo- and/or radiotherapy and cancer-directed surgery. Information regarding whether cancer-directed surgery was recommended by a physician and performed, recommended but not performed, or contraindicated (not recommended) was recorded. Patients were grouped into three socioeconomic status (SES) groups according to median household income—Low (< $40,000–$59,999), Medium ($60,000–$94,999), and High ($95,000–$120,000+).

Descriptive statistics were reported as n and percentages for categorical values. Univariate, between-group comparisons were performed using the chi-square test. Multivariate logistic regression was used to analyze the association between treatment patterns and various patient characteristics. Multivariable Cox regression analysis was performed to examine the association between patient characteristics and overall survival (OS) and cancer-specific survival (CSS). Statistics were carried out using the SEER*Stat software v8.4.5 (NCI, Bethesda, MD) and SPSS v29 (IBM Inc, Armonk, NY). A p-value of less than 0.05 was considered statistically significant.

## Results

### Patient demographics

A total of 10,823 cases were identified during the study period (Table 1). The majority of patients were age 65 years or older (n = 6602, 61%) and male (n = 8407, 78.0%). White patients accounted for 84.9% (n = 9190) of patients. Meanwhile, 8.2% (n = 890) of patients were Black and 8.3% (n = 900) of patients were Hispanic. Among the study population, 23.9% (n = 2591) of patients were classified as low SES, 57.2% (n = 6191) as medium SES, and 18.9% (n = 2041) as high SES.

**Table 1:** Patient demographics

	n = 10,823	
	n	%
Age (65 +)	6602	61
Sex (female)	2416	22.3
Race		
White	9190	84.9
Black	890	8.2
Other	743	6.9
Hispanic	900	8.3
SES		
Low	2591	23.9
Med	6191	57.2
High	2041	18.9

*SES*  Ssocioeconomic status

### Surgical management

48.6% (n = 5260) of patients were recommended surgery for management of their esophageal cancer. Of these patients, 87.2% (n = 4589) underwent surgery. On univariate analysis, female sex was associated with lower rates of surgery recommendations (40.9% vs 50.8%, p < 0.001) and performed surgical intervention (85.0% vs 87.8%, p = 0.023). Similarly, Black patients had lower rates of surgery recommendations (28.2% vs 51.1%, p < 0.001) and surgical intervention (82.5% vs 87.8%, p = 0.002) compared to white patients.

**Fig. 1:**
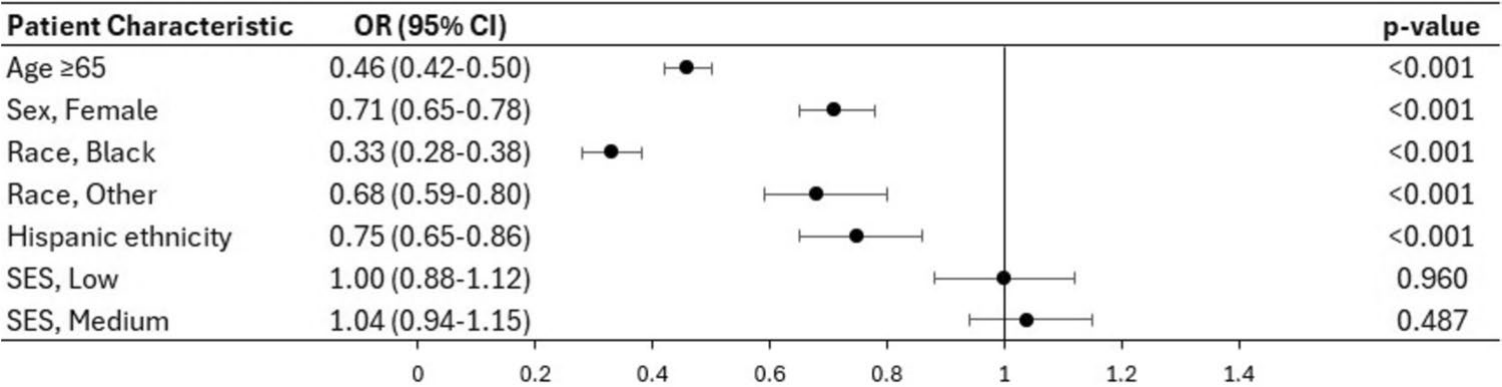
Forest plot based on the results of multivariate analysis of the factors associated with surgery recommendations

**Fig. 2:**
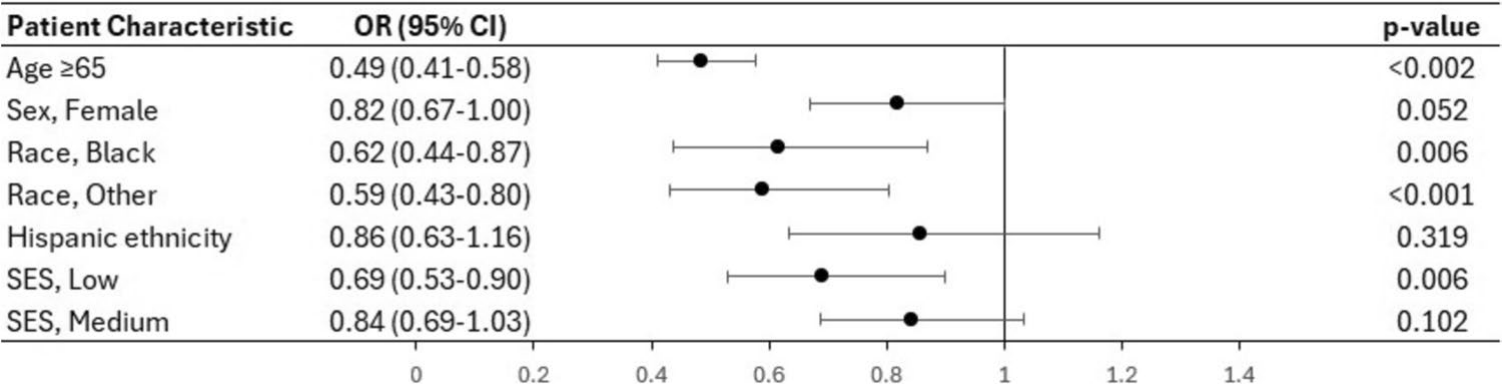
Forest plot based on the results of multivariate analysis of the factors associated with surgical intervention in patients for whom surgery was recommended

On multivariate analysis (Figs. 1 and 2), female gender was independently associated with a lower likelihood of being recommended surgery (OR 0.71, CI 0.65–0.78, p < 0.001). However, there was no difference in the likelihood of undergoing the recommended surgery (OR 0.82, CI 0.67–1.00, p = 0.052). Black patients were less likely to be recommended surgery (OR 0.33, CI 0.28–0.38, p < 0.001) and less likely to undergo surgery (OR 0.32, CI 0.27–0.38, p < 0.001) compared to their White counterparts. Hispanic patients had a lower likelihood of having surgery recommended to them (OR 0.75, CI 0.65–0.86, p < 0.001) but there was no difference in rates of surgical intervention between Hispanic and non-Hispanic patients (OR 0.86, CI 0.63–1.16, p = 0.319). There was no difference between SES groups with regards to the rates of surgery recommendations. However, compared to high SES patients, low SES patients were less likely to undergo surgery when it was recommended (OR 0.69, CI 0.53–0.90, p = 0.006).

### Chemoradiotherapy

Of the patients who underwent surgical intervention, 88.0% (n = 4040) received neoadjuvant therapy (chemotherapy and/or radiation) and 23.3% (n = 1069) received adjuvant therapy. On univariate analysis, female sex (85.1% vs 88.7%, p = 0.005) and Hispanic ethnicity (83.9% vs 88.4%, p = 0.018) were associated with lower rates of neoadjuvant therapy. Female patients also had lower rates of adjuvant therapy (20.4% vs 23.9%, p = 0.027), as did Black patients (15.9% vs 23.6%, p = 0.033) and patients of low SES (Low—20.3% vs Med—23.0% vs High—25.8%, p = 0.02).

**Fig. 3:**
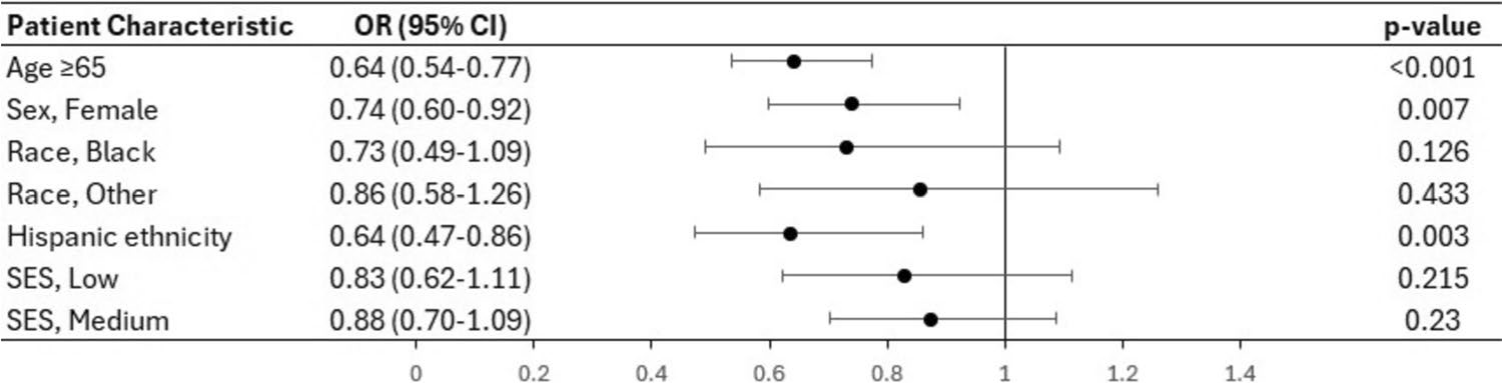
Forest plot based on the results of multivariate analysis of the factors associated with neoadjuvant chemo- or radiation therapy in patients who underwent surgery

On multivariate analysis (Fig. 3), female patients were found less likely to receive neoadjuvant therapy compared to male patients (OR 0.74, CI 0.60–0.92, p = 0.007). In addition, Hispanic patients were less likely to receive neoadjuvant therapy (OR 0.64, CI 0.47–0.86, p = 0.003). There was no difference in rates of neoadjuvant therapy between racial or SES groups.

**Fig. 4:**
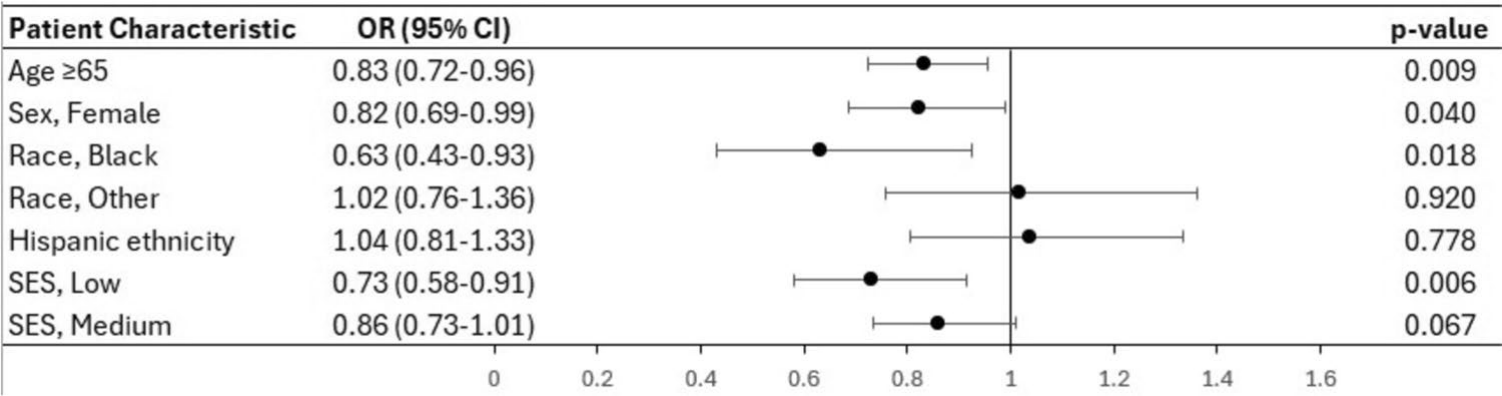
Forest plot based on the results of multivariate analysis of the factors associated with adjuvant chemo- or radiation therapy in patients who underwent surgery

Female patients (OR 0.82, CI 0.69–0.99, p = 0.009), Black patients (OR 0.63, CI 0.43–0.93, p = 0.040), and low SES patients (OR 0.73, CI 0.58–0.91, p = 0.006) were less likely to receive adjuvant chemotherapy and/or radiation (Fig. 4).

### Survival

Despite variations in management, female patients had better overall survival (OS) (HR 0.88, CI 0.83–0.94, p < 0.001) and cancer-specific survival (CSS) (HR 0.91, CI 0.85–0.97, p < 0.001) compared to male patients (Table 2). Meanwhile, Black patients had worse OS (HR 1.32, CI 1.22–1.44, p < 0.001) and CSS (HR 1.31, CI 1.19–1.44, p < 0.001). Similarly, low SES was associated with lower OS (HR 1.15, CI 1.06–1.24, p < 0.001) and CSS (HR 1.17, CI 1.07–1.27, p < 0.001). Medium SES was associated with marginally worse OS (HR 1.07, CI 1.01–1.13, p = 0.034) but equivalent CSS (HR 1.07, CI 1.00–1.14, p = 0.052) compared to high SES.

**Table 2:** Overall and cancer-specific survival analysis

Patient characteristic	Overall survival (OS)		Cancer-specific survival (CS)	
	HR (95% CI)	p-value	HR (95% CI)	p-value
Age ≥ 65	1.36 (1.30–1.43)	< 0.001	1.26 (1.20–1.34)	< 0.001
Sex, female	0.88 (0.83–0.94)	< 0.001	0.91 (0.85–0.97)	0.003
Race, Black	1.32 (1.22–1.44)	< 0.001	1.31 (1.19–1.44)	< 0.001
Race, other	1.01 (0.92–1.12)	0.785	1.04 (0.94–1.16)	0.446
Hispanic ethnicity	0.97 (0.93–1.02)	0.206	0.99 (0.94–1.04)	0.646
SES, Low	1.15 (1.06–1.24)	< 0.001	1.17 (1.07–1.27)	< 0.001
SES, Medium	1.07 (1.01–1.13)	0.034	1.07 (1.00–1.14)	0.052

*SES*  Ssocioeconomic status

## Discussion

The aim of this study was to analyze variations in management for locally advanced esophageal cancer to identify any gender, racial/ethnic, or socioeconomic disparities. Compared to male patients, female patients were less likely to be recommended surgery and to receive neoadjuvant or adjuvant therapy peri-operatively. Non-white patients received fewer recommendations for surgery and were less likely to undergo surgery compared to White patients. Black race and low SES were both associated with lower OS and CSS, whereas female sex was associated with better OS and CSS.

### Sex disparities

Similar to the results presented in this study, prior studies have demonstrated that female patients with esophageal cancer have less access to standard treatments such as chemotherapy, radiation therapy, and surgery [8, 9]. However, data on these disparities affect survival is mixed. Despite differences in management, Kauppila et al. found that female patients had lower mortality rates following esophagectomy for esophageal cancer, consistent with our findings [10]. Furthermore, Bohanes et al. determined that female patients with esophageal squamous cell carcinoma had better CSS compared to male patients [11]. However, other studies report worse survival in female patients given management differences [8]. The underlying reasons for these differences remain unclear but may be attributable to both biological and healthcare access factors. Potential biological factors include hormonal differences and immune response variations that may influence tumor progression and response to treatment [12, 13]. Furthermore, some studies have suggested that male patients present with more comorbidities at the time of cancer diagnosis than female patients, possibly partially explaining differences in survival [14].

### Racial and ethnic disparities

It is well accepted that racial and ethnic disparities in healthcare significantly affect cancer care outcomes, with minority patients experiencing more substantial obstacles to receiving care and being at greater risk of receiving fragmented or poor-quality care [15]. The results of this study align with prior studies which have found that Black and Hispanic patients with esophageal cancer are less likely to receive surgical intervention, which is a critical component of curative treatment for resectable disease [16, 17].

The causes of the racial and ethnic disparities in rates of surgery and survival are multifactorial but may, in part, be due to access to high volume hospitals for esophagectomy and perceptions of care. Utilization of high-volume cancer centers where esophagectomies are usually performed is significantly lower for Black patients compared to White patients [18]. In addition, prior studies have found that patients from racial and ethnic minorities were more likely than White patients to decline surgery for resectable esophageal cancer, reflecting possible differences in trust of the medical system and perceptions of treatment efficacy [19]. This disparity in surgical treatment likely contributes to the observed survival differences between Black and White patients, as surgery is associated with improved long-term survival [20].

### Socioeconomic disparities

Socioeconomic factors play a significant role in treatment disparities, with lower SES patients exhibiting a reduced likelihood of undergoing surgery when recommended. Although there were no significant differences in surgery recommendations across SES groups, the decreased surgical adherence among low SES patients suggests challenges related to healthcare access, affordability, and logistical barriers such as transportation or time off work [21]. In fact, prior research has found that nearly 25% of patients with a history of cancer declined medical care due to costs [22].

These treatment differences appear to negatively impact survival, as found in this study, where low SES patients had significantly worse OS and CSS compared to high SES patients. Previous research has shown that low SES patients are more likely to be diagnosed at advanced stages, have reduced access to high-quality oncology care, and experience greater treatment delays, all of which contribute to poorer outcomes [23, 24].

### Limitations

While this study provides valuable insights into disparities in the management of locally advanced esophageal cancer, several limitations should be considered. First, this study relies on retrospective data, which is subject to inherent biases and potential inaccuracies in coding. Additionally, information regarding confounders, such as patient comorbidities, nutritional status, candidacy for surgery, treatment preferences, and hospital-level factors, are not captured in the SEER database and could not be adjusted for, though they may influence treatment decisions and outcomes. Finally, while we adjusted for SES, our measures may not fully capture individual-level economic barriers to care.

## Conclusions

Despite its limitations, this study highlights significant gender, racial, ethnic, and socioeconomic disparities in the management of locally advanced esophageal cancer. Female patients, despite their survival advantage, were less likely to receive aggressive treatment, while Black patients had reduced surgical recommendations and worse survival outcomes. Hispanic patients were less likely to be recommended surgery and to receive neoadjuvant surgery. Socioeconomic disparities further compounded these inequities, particularly in treatment adherence and survival. Addressing these disparities requires a multifaceted approach, including provider education to mitigate implicit biases, policy reforms to improve healthcare access, and community-based interventions to support patients facing socioeconomic barriers. Future research should focus on identifying actionable strategies to promote equitable treatment and improve outcomes for all patients with esophageal cancer.

## References

[1] Then EO, Lopez M, Saleem S et al. (2020) Esophageal cancer: an updated surveillance epidemiology and end results database analysis. World J Oncol 11:55–64. 10.14740/wjon1254 32284773 10.14740/wjon1254 PMC7141161

[2] Ke J, Xie Y, Huang S et al (2024) Comparison of esophageal cancer survival after neoadjuvant chemoradiotherapy plus surgery versus definitive chemoradiotherapy: a systematic review and meta-analysis. Asian J Surg 47:3827–3840. 10.1016/j.asjsur.2024.02.099 38448293 10.1016/j.asjsur.2024.02.099

[3] Huang R, Qiu Z, Zheng C et al (2022) Neoadjuvant therapy for locally advanced esophageal cancers. Front Oncol 12:734581. 10.3389/fonc.2022.734581 35463306 10.3389/fonc.2022.734581 PMC9021527

[4] Ran X, Zeng H, Zheng R et al (2024) Geographic, sex and socioeconomic disparities in esophageal cancer incidence in China: a population-based study. Int J Cancer 154:477–487. 10.1002/ijc.34730 37728072 10.1002/ijc.34730

[5] Geng CX, Gudur AR, Radlinski M et al (2023) Socioeconomic disparities affect outcomes in early-stage esophageal adenocarcinoma: a SEER analysis. Clin Gastroenterol Hepatol 21:2797-2806.e6. 10.1016/j.cgh.2023.02.011 36858145 10.1016/j.cgh.2023.02.011

[6] Tran P, Taylor T, Klempner S, Zell J (2017) The impact of gender, race, socioeconomic status, and treatment on outcomes in esophageal cancer: a population-based analysis. J Carcinog 16:3. 10.4103/jcar.JCar_4_17 28974922 10.4103/jcar.JCar_4_17 PMC5615860

[7] Shah MA, Kennedy EB, Alarcon-Rozas AE et al (2023) Immunotherapy and targeted therapy for advanced gastroesophageal cancer: ASCO guideline. J Clin Oncol 41:1470–1491. 10.1200/JCO.22.02331 36603169 10.1200/JCO.22.02331

[8] Baumrucker C, Franceschi D, Livingstone AS, Macedo FI (2021) Impact of gender on treatment and survival of patients with esophageal cancer in the United States. J Clin Oncol 39:173–173. 10.1200/JCO.2021.39.3_suppl.17333290127

[9] Kalff MC, Wagner AD, Verhoeven RHA et al (2022) Sex differences in tumor characteristics, treatment, and outcomes of gastric and esophageal cancer surgery: nationwide cohort data from the Dutch Upper GI Cancer Audit. Gastric Cancer 25:22–32. 10.1007/s10120-021-01225-1 34365540 10.1007/s10120-021-01225-1 PMC8732809

[10] Kauppila JH, Wahlin K, Lagergren P, Lagergren J (2019) Sex differences in the prognosis after surgery for esophageal squamous cell carcinoma and adenocarcinoma. Int J Cancer 144:1284–1291. 10.1002/ijc.31840 30168595 10.1002/ijc.31840

[11] Bohanes P, Yang D, Chhibar RS et al (2012) Influence of sex on the survival of patients with esophageal cancer. J Clin Oncol 30:2265–2272. 10.1200/JCO.2011.38.8751 22585694 10.1200/JCO.2011.38.8751 PMC3397720

[12] Yan H, Huang J, Li Y, Zhao B (2024) Sex disparities revealed by single-cell and bulk sequencing and their impacts on the efficacy of immunotherapy in esophageal cancer. Biol Sex Differ 15:22. 10.1186/s13293-024-00598-z 38491510 10.1186/s13293-024-00598-z PMC10941500

[13] Wang BJ, Zhang B, Yan SS et al (2016) Hormonal and reproductive factors and risk of esophageal cancer in women: a meta-analysis. Dis Esophagus 29:448–454. 10.1111/dote.12349 25809699 10.1111/dote.12349

[14] Koppert LB, Janssen-Heijnen MLG, Louwman MWJ et al (2004) Comparison of comorbidity prevalence in oesophageal and gastric carcinoma patients: a population-based study. Eur J Gastroenterol Hepatol 16:681–688. 10.1097/01.meg.0000108331.52416.f1 15201582 10.1097/01.meg.0000108331.52416.f1

[15] Esnaola NF, Ford ME (2012) Racial differences and disparities in cancer care and outcomes: where’s the rub? Surg Oncol Clin N Am 21:417–437, viii. 10.1016/j.soc.2012.03.012 22583991 10.1016/j.soc.2012.03.012 PMC4180671

[16] Savitch SL, Grenda TR, Scott W et al (2021) Racial disparities in rates of surgery for esophageal cancer: a study from the national cancer database. J Gastrointest Surg 25:581–592. 10.1007/s11605-020-04653-z 32500418 10.1007/s11605-020-04653-z

[17] Ricardo J, Alkayali T, Shridhar R et al (2024) Esophageal cancer in Hispanics: a demographic analysis of the National Cancer Database. J Gastrointest Surg 28:1126–1131. 10.1016/j.gassur.2024.05.010 38740256 10.1016/j.gassur.2024.05.010

[18] Rehmani SS, Liu B, Al-Ayoubi AM et al (2018) Racial disparity in utilization of high-volume hospitals for surgical treatment of esophageal cancer. Ann Thorac Surg 106:346–353. 10.1016/j.athoracsur.2018.03.042 29684373 10.1016/j.athoracsur.2018.03.042

[19] Patel VR, Liu M, Snyder RA et al (2024) Trends in racial and ethnic differences in declined surgery for resectable malignancies in the United States. Ann Surg. 10.1097/SLA.0000000000006516 39225400 10.1097/SLA.0000000000006516 PMC12163911

[20] Chen M-F, Chen P-T, Lu M-S et al (2017) Survival benefit of surgery to patients with esophageal squamous cell carcinoma. Sci Rep 7:46139. 10.1038/srep46139 28383075 10.1038/srep46139 PMC5382669

[21] Bourgeois A, Horrill T, Mollison A et al (2024) Barriers to cancer treatment for people experiencing socioeconomic disadvantage in high-income countries: a scoping review. BMC Health Serv Res 24:670. 10.1186/s12913-024-11129-2 38807237 10.1186/s12913-024-11129-2 PMC11134650

[22] Zafar SY, Peppercorn JM, Schrag D et al (2013) The financial toxicity of cancer treatment: a pilot study assessing out-of-pocket expenses and the insured cancer patient’s experience. Oncologist 18:381–390. 10.1634/theoncologist.2012-0279 23442307 10.1634/theoncologist.2012-0279 PMC3639525

[23] Chen KA, Strassle PD, Meyers MO (2021) Socioeconomic factors in timing of esophagectomy and association with outcomes. J Surg Oncol 124:1014–1021. 10.1002/jso.26606 34254329 10.1002/jso.26606 PMC10151060

[24] Wang N, Cao F, Liu F et al (2015) The effect of socioeconomic status on health-care delay and treatment of esophageal cancer. J Transl Med 13:241. 10.1186/s12967-015-0579-9 26205792 10.1186/s12967-015-0579-9 PMC4511992

